# Assessing mobile phone access, usage, and willingness among women to receive voice message-based mobile health intervention to improve antenatal care attendance in district Thatta, Sindh, Pakistan

**DOI:** 10.1186/s12978-020-00956-1

**Published:** 2020-07-06

**Authors:** Anam Shahil Feroz, Naureen Akber Ali, Sarah Saleem

**Affiliations:** 1 Department of Community Health Sciences, The Aga Khan University, Stadium Road, PO Box 3500, Karachi, 74800 Pakistan; 2 School of Nursing and Midwifery, The Aga Khan University, Stadium Road, PO Box 3500, Karachi, 74800 Pakistan

**Keywords:** Mobile phone, Access, Usage, Willingness, Voice message-based mHealth intervention, Antenatal care attendance, District Thatta

## Abstract

**Background:**

Pakistan has one of the highest maternal mortality ratios worldwide at 276/100,000 live births and only 51% percent of women receive four or more ANC visits. This means that there are missed opportunities for almost half of the women who were not able to seek the recommended antenatal visits. In Thatta district, the maternal mortality ratio is estimated at 313/100,000 live births. Various studies reported that mHealth interventions have proven to be effective to improve antenatal care and postnatal care services. However, the feasibility and effectiveness of mobile health interventions to increase uptake of preventive maternal healthcare services among pregnant women in different settings may be different due to differing patient demographics, cultural diversity, environmental and behavioral factors, availability and accessibility to mobile phones, and budgetary constraints. Prior to implementing a similar intervention in Thatta District, it is crucially important to assess the mobile phone access, usage and willingness among women to receive voice-message based mHealth intervention to improve antenatal care attendance.

**Methods:**

A cross-sectional quantitative study will be used to assess mobile phone access, usage and willingness among women to receive voice-message based mHealth intervention to improve antenatal care attendance in district Thatta. The study will be conducted in Thatta district of Sindh province. Married women of reproductive age (MWRA), who are residing in selected villages of Mirpur Sakro and willing to participate will be included in the study. Multistage sampling technique will be used to recruit the 415 study participants. A structured questionnaire has been designed on Epicollect to collect data from 415 women. Data will be analyzed using IBM SPSS Statistics version 23, with a level of significance as < 0.05.

**Discussion:**

This research project will provide invaluable information on the current access, usage of mobile phones among women of district Thatta and their willingness to receive voice messages to improve the antenatal care services. The study will also highlight demographic, sociocultural and economic factors associated with women willingness and readiness to receive voice messages regarding antenatal care.

## Plain English summary

One out of 140 Pakistani women faces a lifetime risk of maternal death. Most women die because of complications that occur during pregnancy, at delivery, and during the postnatal period. Evidence suggests that antenatal care (ANC) from a skilled provider is important to timely identify and manage preventable maternal morbidities. Several strategies have proven to be effective to improve antenatal care and postnatal care services. However, the effectiveness of these strategies to increase uptake of ANC attendance among pregnant women in different settings may be different due to several factors. Prior to applying a similar intervention in Thatta District, it is crucially important to measure the mobile phone access, usage, and willingness among women to receive voice-message based mHealth intervention to improve antenatal care attendance. A survey will be conducted to assess mobile phone access, usage, and willingness among women to receive voice-message based mHealth intervention to improve antenatal care attendance in district Thatta. This research project will provide invaluable information on the current access, usage of mobile phones among women of district Thatta and their willingness to receive voice messages to improve the antenatal care services.

## Background

In developing countries, women face a lifetime risk of maternal death of one in 160, as compared with 1 in 3700 for women living in developed countries [1]. Pakistan has one of the highest maternal mortality ratios worldwide at 276/100,000 live births [2]. One out of 140 Pakistani women faces a lifetime risk of maternal death [3]. Most women die because of complications that occur during pregnancy, at delivery, and during the postnatal period [4]. Evidence suggests that antenatal care (ANC) from a skilled provider is important to timely identify and manage preventable maternal morbidities such as preeclampsia, eclampsia, antepartum hemorrhage, obstructed labor, postpartum hemorrhage and puerperal sepsis, which are contributing to nearly 70% of all maternal deaths [2]. ANC interventions have proven to be key health interventions to decrease maternal mortality in LMICs, such as Tanzania and Ethiopia [5, 6]. Most importantly, there is need for reinforcement of prevailing evidence-based practices that include World Health Organization (WHO) recommended number of ANC visits (minimum of four ANC visits) to improve maternal health outcomes [7].

The Pakistan Demographic and Health survey (PDHS) 2017–18 shows that only 51% percent of women received four or more ANC visits [8]. This means that there are missed opportunities for almost half of the women who were not able to seek the recommended antenatal visits. In Thatta district, the maternal mortality ratio is estimated at 313/100,000 live births. This estimate is based on a Maternal and Newborn Health Registry (MNHR), which is maintained by Global Network for Women’s and Children’s Health Research Network (GN). A recent trend analysis of ANC showed that the percentages of women receiving at least four ANC visits in Thatta is substantially lower (40%) [9]. To meet the targets of the United Nations’ Sustainable Development Goal 3 by 2030 (maternal mortality ratio < 70/100,000 live births) [10], novel and innovative strategies are required to decrease the rate of maternal mortality by improving the uptake of ANC services. An apparent and accessible strategy to meet this goal is to utilize the potential of simple mobile phones to increase the number of ANC visits (at least 4) among antenatal women.

Various studies reported that mHealth interventions, particularly those delivered through SMS and voice calls, are associated with improved utilization of preventive maternal healthcare services, including uptake of recommended ANC and PNC services. In Njoro Division, a randomized controlled trial evaluated the impact of mobile telephone support on antenatal attendance. A group of 191 pregnant women were regularly given advice and prompts regarding pregnancy care and scheduled antenatal visits through mobile phone; whereas the other groups of 206 pregnant women were provided usual care to continue antenatal visit. Positive association was found among women in intervention group and the number of ANC visits (96.4% in intervention group and 92.3% in the control group, *P* value: 0.002) [11]. A pragmatic cluster randomized controlled trial was conducted in primary healthcare facilities of Zanzibar. The primary outcome measure of the trial was four or more ANC visits. The SMS intervention was related with an improvement in ANC visits in the intervention group. In the intervention group, 44% of women attained four or more ANC visits versus 31% in the comparison group (OR, 2.39; 95% CI 1.03–5.55) [12]. In a rural area of Tamil Nadu, India, a pre-post study was conducted to evaluate whether mobile text messaging service is a feasible mode of raising knowledge level regarding Maternal and Child Health (MCH) services. Data was obtained using a questionnaire in three phases; a) baseline assessment, b) intervention: MCH related messages were sent, c) end line assessment. It was found out that 45 (37.5%) individuals knew about minimum number of antenatal visits during pregnancy after receiving text messages, as compared to 12 (10%) individuals before receiving text messages (*P* value < 0.05, 95% CI: 0.16–0.38) [13]. Three systematic reviews have been conducted on using mHealth applications for improving antenatal and postnatal care in low and middle income countries. All of reviews have reported that mHealth interventions have proven to be effective to improve antenatal care and postnatal care services, especially those that are aimed at changing behavior of pregnant women through SMS and voice messages [14–16].

However, the feasibility and effectiveness of mobile health interventions to increase uptake of preventive maternal healthcare services among pregnant women in different settings may be different due to differing patient demographics, cultural diversity, environmental and behavioral factors, availability and accessibility to mobile phones, and budgetary constraints. Prior to implementing a similar intervention in Thatta District, it is crucially important to assess the mobile phone access, usage and willingness among women to receive voice-message based mHealth intervention to improve antenatal care attendance.

### Scope and objectives

- To assess the access and usage of mobile phone among MWRA in district Thatta, Karachi
- To determine the willingness of MWRA to receive voice-message based mHealth intervention to improve antenatal care attendance in district Thatta, Karachi

### Operational definitions

- Mobile phone access: Access to basic mobile phone or smart phone, mobile phone ownership (self or shared)
- Mobile phone usage: quantifies the extent to which a person uses a phone, or categorizes the types of uses and situations in which use occurs
- Willingness for mHealth intervention: Women willing to receive voice message to improve ANC attendance. If yes, what are the preferences for language, time to receive voice message etc. If no, what are the barriers or reasons for poor willingness

### Hypothesis

Married women of reproductive age in district thatta, who have access to simple mobile phones, would be willing to receive voice message to improve antenatal care attendance.

## Methodology

### Study design

A cross-sectional quantitative study will be used to assess mobile phone access, usage and willingness among women to receive voice-message based mHealth intervention to improve antenatal care attendance in District Thatta.

### Study setting

Thatta district of Sindh province, lies at the coast of Arabian Sea and is situated about 100 km from the city of Karachi. The total population of district Thatta is about 1 million, which is primarily a rural area (82%). Most people of Thatta speak Sindhi language (87%). About 76% of the population are concentrated in two talukas (sub-districts, Thatta and Mirpur Sakro) of the total of four talukas in the district. The study will be conducted in Mirpur Sakro taluka of district thatta, where Community Health Sciences Department of Aga Khan University has recently established a Rural Health Program (RHP). Mirpur Sakro, is the second biggest taluka of the district in terms of population and has 14 union councils, and 2 town committees. The RHP program is running in 125 villages. The data will be collected from 20 purposively selected villages out of 125 villages. The villages have been divided into three stratas, based on their road distance from health facility (Taulka hospital). The three strata are defined as follows: (i) Villages situated less than 5kms from the health facility (ii) Villages that are 5-10kms away and; (iii) Villages that are more than 10kms away from the health facility.

### Study subjects

Married women of reproductive age (MWRA), who are residing in selected villages of Mirpur Sakro and willing to participate will be included in the study.

### Sample size

**Fig. 1:**
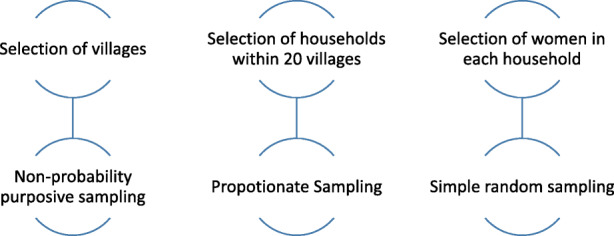
Sampling technique

Multistage sampling technique will be used to recruit the participants (Fig. 1). According to our best knowledge, we could not find any studies conducted to determine the mobile phone usage and willingness among women to receive SMS text message–based mHealth interventions to improve ANC services in Pakistan, although the mobile phone access in Pakistani women is 39.2%, according to Pakistan demographic and health survey (PDHS) [8]. Therefore, we assumed that 50% of women use mobile phone and are willing to receive voice-message based mHealth intervention for improving ANC services. The maximum sample size is 384, which was calculated using the proportion of women who have mobile phone and are willing to receive voice-message based mHealth intervention for improving ANC services. Considering a 10% nonresponse rate, we calculated the final sample size to be 415. A non-probability purposive sampling will be used to select 415 MWRA, from the households due to lack of availability of household line listing. From each of the 20 villages, households will be selected using proportionate sampling to complete sample size of 415. From beach household, one women will be randomly selected for the purpose of this survey.

### Data collection tool

A structured questionnaire will be designed to assess mobile phone access, usage and willingness among women to receive voice-based mHealth intervention to improve ANC services. The survey instrument will be developed in English, which will be underwent forward and backward translation to ensure semantic consistency (English to Sindhi then English). The instrument will be in three main sections such as: Socio-demographic characteristics, ownership, knowledge and usage of mobile phone and willingness for receiving voice message for antenatal care attendance. Before the data collection, a pilot testing of questionnaire will be conducted on fifty women), belonging to other talkuka. The necessary modifications will be made on the basis of pretest findings.

### Data collection methodology

Epicollect will be used to collect data from 415 women. Epicollect5 is a free web-based tool that enables to collect customized data (including location and media) using a mobile device. The data collection form will be created on the Epicollect 5 app, which can be opened on smartphone by a data collector to collect data in the field (either online or offline). The real-time data can be viewed and downloaded on excel sheet for analysis. Three data collectors will be hired and trained for data collection. One week training of data collectors will be conducted to brief them about study goals and objectives, research ethics (taking informed consent), study questionnaire and use of Epicollect 5 app. The PI and field supervisor will monitor the field activities to ensure the transparency and quality of data collection. Quality of data will also assessed through random checks by the principal Investigator.

### Ethical considerations

- Consent of participation & publication will be taken from the study participants.
- Ethical approval has been taken from Aga Khan University Ethical Review Committee
- Participants will be assured that the given information will be kept confidential and only be used for research purpose.

### Data analysis

Data will be analyzed using IBM SPSS Statistics version 23, with a level of significance as < 0.05

- Descriptive: Frequencies, means, and SD will be calculated to describe the variables. Demographic and mobile phone usage data will be compared between respondents belonging to different villages.
- Inferential: Chi-square and binary logistic regression models will be used to identify factors associated with willingness to use mobile phones for improving antenatal care attendance. Multiple logistic regression will be performed to determine adjusted odds ratio (AOR) for the willingness to use mobile phones for antenatal care attendance; the adjusted variables included residence, ability to read, ownership of mobile phone, routine use of mobile, age group, and duration of mobile phone use.

## Discussion

This research project will provide invaluable information on the current access, usage of mobile phones among women of district Thatta and their willingness to receive voice messages to improve the antenatal care services. The study will also highlight demographic, sociocultural and economic factors associated with women willingness and readiness to receive voice messages regarding antenatal care. These insights will guide researchers on how mHealth programs can be tailored to local context, through developing cultural-sensitive messages in local languages, and following societal norms (sending messages on specific time of the day), to ensure successful implementation of such programs. These insights will support decisions on introducing tailored mHealth programs in Thatta and other parts of Pakistan to improve the antenatal care services. While mHealth programs has been proven effective for various chronic diseases and public health issues, it is a novel idea for supporting pregnant individuals for preventative maternal healthcare services, with very few previous studies in area. In this study, if willingness among women is captured towards mHealth intervention, our team will work to implement voice message-based mHealth intervention to improve ANC attendance among women of district.

## Supplementary information

**Additional file 1.**

## Data Availability

Materials described in this paper pertain to the study protocol only and there are no raw data reported. The datasets will be collected and analyzed and can be made available from the corresponding author on reasonable request.

## Abbreviations

ANC: Antenatal care
AOR: Adjusted odds ratio
GN: Global Network
MCH: Maternal Child Health
MNHR: Maternal Neonatal Health Registry
MWRA: Married women of reproductive age
PDHS: Pakistan Demographic & Health Survey
RHP: Rural Health Program
SMS: Short Message Service
WHO: World Health Organization
