# Supplementary material for: Assessing mobile phone access, usage, and willingness among women to receive voice message-based mobile health intervention to improve antenatal care attendance in district Thatta, Sindh, Pakistan
Source: Reprod Health. 2020 Jul 6;17:104. doi: 10.1186/s12978-020-00956-1 (PMC7336476; doi:10.1186/s12978-020-00956-1)
Supplement: Supplementary file 1 — Additional file 1. [file 12978_2020_956_MOESM1_ESM.docx]

The instrument will be in three main sections such as: Socio-demographic characteristics, ownership, knowledge and usage of mobile phone and willingness for receiving voice message for antenatal care attendance.

**Date of Survey: _____________ Data Collector (Name): __________**

**Section 1: Socio Demographic Information:**

1. Number of members living in your house: ________
2. Number of adults (18 years and above): ______________
   1. Male: ______________
   2. Female: _________________
3. Number of children (17 years and below) : __________
   1. Male: ______________
   2. Female: ____________
4. Age: ____________
5. Education level:
   1. No formal
   2. Primary school
   3. secondary school
   4. Intermediate/high school
   5. diploma
   6. Vocational training
   7. bachelor
   8. Post –graduate/ masters
6. Employment status
   1. Employed for wages.
   2. Employed-monthly income
   3. Unemployed.
   4. Self-employed
   5. Retired
   6. Student
   7. A homemaker
7. Marital Status:
   1. Married living with spouse
   2. Married but separated
   3. Divorced
   4. widow
8. Language (mother tongue):_______________
9. Languages known:
   1. (To speak only)___________________________
   2. (To read and write)________________________

**Section 2: Ownership, knowledge and usage of mobile phone**

1. Number of mobile phones owned in the entire household:
   1. By the males: ________
   2. By the females: _______
2. Do you own a mobile phone?
   1. Yes [If Yes, skip to question 14 ]
   2. No [If No, complete question 12-13 ]
3. If no, do you share a mobile phone with any family member?
   1. Yes
   2. No
4. If ‘Yes’ shared with:
   1. Husband
   2. Sister-in-law
   3. Mother-in-law
   4. Other __________
5. Do you routinely use mobile phones?
   1. Yes
   2. No
6. Type of mobile phone you use: [either shared or personal]
   1. Basic mobile phone
   2. Smart phone
7. Amount of money you spent on the mobile phones per month (in PKR):___________
8. Do you use the mobile phone to send text messages (SMS)?
   1. Yes
   2. No
9. If ‘yes’, how often do you use to send/ receive text messages?
   1. Send an SMS: ______per day/___ per week
   2. Receive an SMS: ______per day/___ per week
10. Do you use mobile phone to call?
    1. Yes
    2. No
11. If ‘yes’, how often do you for calls:
    1. Call others: ________per day/___ per week
    2. Receive calls: _______per day/___ per week
12. What prevents you from using your mobile phone? (Multiple response possible)
    1. Cannot afford
    2. No network available in my area
    3. Do not need it
    4. Do not know how to use the mobile phone
    5. Not allowed to use a mobile phone
    6. Other: Please specify________
13. What other functions/applications do you use?
    1. Whatsapp
    2. Facebook
    3. Instagram
    4. YouTube
    5. Others ___________
14. Since when have been using a mobile phone?
    1. _________ Years/ ________months.
15. Do you use your mobile for setting an alarm?
    1. Yes
    2. No
16. What purpose do you use the alarm for? (Multiple answers possible)
17. As a wake up call
18. To remind me of events
19. As a reminder for medication
20. To remind for prayers
21. Other________
22. For what else do you use the mobile phone? (Multiple answers possible)
23. For listening to radio/music
24. For playing games
25. To use the camera
26. To access the internet
27. Others uses________

**Section 3: Willingness for receiving voice message for antenatal care attendance**

1. Would you like to receive general health advice on your mobile phone?
2. Yes
3. No
4. Would you like to receive alerts about your next antenatal visit on your phone?
5. Yes
6. No
7. If yes, when would you like to receive the reminder?
8. One day before the date of antenatal visit
9. On the day of antenatal visit
10. One week earlier to antenatal visit
11. Would you like to receive information on antennal care?
12. Yes
13. No
14. If yes, what topics would you like receiving information on?
15. Nutrition
16. Healthy life style
17. Pregnant woman’s health
18. Mother and Child health
19. Vaccinations
20. How often would you like to receive this information?
21. Daily
22. Once a week
23. Once a month
24. Would you like to receive reminders for antenatal vaccination during your pregnancy?
25. Yes
26. No
27. If yes, when would you like to receive the reminder?
28. One day before the date of antenatal vaccination
29. On the day of antenatal vaccination
30. One week earlier to antenatal vaccination
31. Would you like to provide your mobile number to doctor so that he or she may contact you?
32. Yes
33. No
34. At what time of the day you would like to receive reminders or general messages on antenatal care?
35. Morning
36. Afternoon
37. Evening
38. Night
39. Why would you like to receive information on this specific time of the day?

________________________

1. In which language, would you like to receive all the information?
2. Language (first preference):_________
3. Language (second preference): _____________
4. What type of mHealth intervention you would like to receive?
5. SMS
6. Voice clip
7. Voice call
8. Video message
9. Whatsapp voice message
10. Other ___________
